# Burnout, Compassion Satisfaction, and Intent to Quit Among Long-Term Care Nursing Assistants in the Time of COVID-19

**DOI:** 10.1093/geroni/igab046.2666

**Published:** 2021-12-17

**Authors:** Mallory Richert

**Affiliations:** Xavier University, Cincinnati, Ohio, United States

## Abstract

The COVID-19 pandemic has greatly exacerbated the stress and burden of those employed in long-term care (LTC) facilities due to staff shortages, increased risks on the job, and ever-changing COVID-19 protocol requirements. This study examines potential differences in pre-COVID-19 and current COVID-19 LTC facility employed nursing assistants on burnout, compassion satisfaction, job satisfaction, and intent to quit. The sample included 81 nursing assistants employed in LTC facilities across the United States, with data collected prior to (n= 42) and during COVID-19 related shutdowns (n= 39). Participants completed the Professional Quality of Life Scale 5 (ProQOL 5), a single-item self-report measure of job satisfaction, and a two-item self-report measure of intent to quit their current employment. Nursing assistants during COVID-19 reported a higher level of burnout and lower level of compassion satisfaction than nursing assistants Pre-COVID-19. However, there were no differences in job satisfaction or intent to quit. The results suggest there may be additional factors that influence an individual’s decision to remain employed above and beyond the impacts of burnout and compassion satisfaction that may be unique to the caring professions. Future research might investigate factors that influence an individual’s decision to remain employed as a nursing assistant during periods of increased stress and burnout. Additionally, the impact of COVID-19 related stress added to the already high levels of stress and burnout on nursing assistants calls for further attention and research devoted to psychological support of LTC staff during crisis and normal times.

